# Commentary: APP as a Mediator of the Synapse Pathology in Alzheimer's Disease

**DOI:** 10.3389/fncel.2018.00150

**Published:** 2018-05-31

**Authors:** An Schreurs, Amira Latif-Hernandez, Alice Uwineza

**Affiliations:** ^1^Brain & Cognition, Faculty of Psychology and Educational Sciences, KU Leuven – University of Leuven, Leuven, Belgium; ^2^Neurology & Neurological Sciences, Stanford University School of Medicine, Stanford, CA, United States; ^3^Department of Bioscience, Durham University, Durham, United Kingdom

**Keywords:** Alzheimer's disease, amyloid-beta, amyloid precursor protein, electrophysiology, oligomers, synaptic plasticity, tau, transgenic mouse model

Alzheimer's disease (AD) is the most common neurodegenerative disorder, but despite decades of extensive research, a disease-modifying therapy is still lacking. AD is a multifactorial disease that occurs in familial and sporadic forms, but is always accompanied by neurotoxic accumulations of amyloid and tau proteins. The deposition of amyloid was postulated to be central for AD pathogenesis in the “amyloid cascade hypothesis” (Hardy and Higgins, 1992) that still dominates the field. In its latest update, soluble oligomers are considered the most toxic species (reviewed by Benilova et al., 2012; Ferreira et al., 2015). These secreted oligomers interact with many synaptic receptors (e.g., glutamate, insulin, adrenergic, and neurotrophin receptors), hereby aberrantly activating distinct signaling cascades, resulting in disrupted calcium homeostasis, excitotoxicity, neurodegeneration, mitochondrial dysfunction, and major synaptic dysfunction (Ferreira et al., 2015). The damaging effects of oligomers at synapses are believed to initiate AD, making synaptic readouts especially useful to investigate its early phases (Selkoe, 2002). A common method to study synaptic function is *in vitro* induction of long-term potentiation (LTP), a form of synaptic plasticity, the cellular basis of learning and memory.

The source of amyloid is the amyloid precursor protein (APP), which belongs to a small family of transmembrane proteins together with amyloid precursor-like proteins (APLP) 1 and 2. APP can undergo distinct secretase-mediated cleavages, following either the “non-amyloidogenic” or “amyloidogenic” pathway. The first serves a range of important physiological functions, including regulation of transcription and synaptic plasticity (reviewed by Müller et al., 2017). In contrast, the amyloidogenic pathway releases amyloid-beta (Aβ) peptides of different lengths that form the harmful oligomers. Mutations in genes related to APP processing and in the APP gene itself cause familial AD (Van Cauwenberghe et al., 2016), but it is unclear whether APP is merely the Aβ precursor, or whether it plays additional pathological roles in AD.

In this commentary, we want to highlight two recent papers that addressed this question using overlapping and complementary methods: Puzzo et al. (2017) and Wang et al. (2017). Both groups independently performed electrophysiological recordings on hippocampal slices from APP-KO mice, and examined whether the absence of APP alters the disruptive effect of applied Aβ oligomers on synaptic plasticity. Their results convey exactly the same message: APP-KO mice are spared from the severe LTP deficits caused by oligomers in wildtype mice. In addition, Wang et al. (2017) reported that the presynaptic release probability and excitation/inhibition balance are elevated by oligomers in wildtype, but not in APP-KO mice. These findings could reflect a general pathological mechanism with network-wide effects (Palop and Mucke, 2016) and are in line with a previously reported role for APP in regulating GABAergic inhibition (Wang et al., 2014). Wang et al. (2017) further showed that APP is required for Aβ's co-localization with pre- and postsynaptic markers, confirming a toxic Aβ-APP interaction.

On the other hand, Puzzo et al. (2017) investigated whether the synaptotoxic effects of tau, the other key player in AD, are likewise APP-dependent. They found that this is indeed the case, and moreover, that both Aβ and tau oligomers can bind to APP and even require APP for efficient internalization (Puzzo et al., 2017). Importantly, this discloses APP as a shared interaction partner between amyloid and tau. To examine whether these electrophysiological and molecular findings are reflected in cognitive measures, Puzzo et al. (2017) applied contextual fear conditioning and radial arm water maze, which interestingly revealed that knockout of APP also prevents oligomer-induced deficits in associative and spatial memory.

Taken together, the results from Wang et al. (2017) and Puzzo et al. (2017) convincingly show that APP is an important mediator of oligomer toxicity. Nevertheless, since APP has so many synaptic functions, it cannot be completely excluded that constitutive APP-KO results in structural synaptic changes or altered signaling pathways which could alternatively explain the insensitivity to oligomers. Furthermore, both groups used young APP-KO mice which display normal LTP, while these mice do develop LTP deficits at older ages (Müller et al., 2017). Although the application of oligomers to young mice could model relevant pathological processes of early AD, it seems essential to repeat the experiments with mice of a more advanced age, which is more relevant for human AD and takes possible age-related alterations of synaptic properties into account (Mattson and Magnus, 2006).

Another important open question is whether similar results would have been obtained using mice with a more acute, conditional APP reduction, such as a tamoxifen-inducible APP-KO line (Callahan et al., 2017), especially in light of the translational potential for APP-targeted therapy in patients. Similarly, it remains to be investigated whether other members of the APP family are also involved in oligomer toxicity, and likewise offer any therapeutic potential. Since only APP can release Aβ peptides, but APP and APLP2 are functionally redundant (Fanutza et al., 2015; Müller et al., 2017), it may be tempting to upregulate APLP2 while APP is downregulated, to compensate for APP's loss of function without affecting Aβ production and toxicity.

The new pathological role for APP has implications for studies using transgenic AD mouse models, which typically overexpress human APP (recent overview in Jankowsky and Zheng, 2017). Given that APP is directly involved in oligomer toxicity, models with a more physiological APP expression, e.g., humanized knock-ins, likely have higher construct validity.

In conclusion, the studies by Wang et al. (2017) and Puzzo et al. (2017) lead to a model wherein APP acts as a central pre- and postsynaptic linking molecule that mediates both Aβ- and tau-induced synaptic and behavioral deficits. The results strongly suggest that APP should be considered as therapeutic target, to counteract the toxic (inter)actions of Aβ and tau at the synapse and preserve crucial neuronal networks. Nevertheless, the findings in the hippocampus of APP-KO mice will need to be successfully translated to other AD-affected brain regions and to human patients. The huge number of failed clinical trials and the news of top pharma companies withdrawing from AD research are extremely worrying, but let's continue to join forces to find that much needed cure.

## Author contributions

AS conceived the study and wrote the original draft with critical and substantial input from AU. AL-H provided valuable ideas and revisions. All authors reviewed relevant literature, participated in discussions, and approved the final version of the manuscript.

### Conflict of interest statement

The authors declare that the research was conducted in the absence of any commercial or financial relationships that could be construed as a potential conflict of interest.
